# Comparing the Use of High- to Low-Cost Black Carbon and Carbon Dioxide Sensors for Characterizing On-Road Diesel Truck Emissions

**DOI:** 10.3390/s20236714

**Published:** 2020-11-24

**Authors:** Rebecca A. Sugrue, Chelsea V. Preble, Thomas W. Kirchstetter

**Affiliations:** 1 Department of Civil and Environmental Engineering, University of California, Berkeley, CA 94720, USA; rasugrue@berkeley.edu (R.A.S.); cvpreble@berkeley.edu (C.V.P.); 2 Energy Technologies Area, Lawrence Berkeley National Laboratory, Berkeley, CA 94720, USA

**Keywords:** truck emissions, black carbon, emission factors, low-cost sensors

## Abstract

The exhaust plume capture method is a commonly used approach to measure pollutants emitted by in-use heavy-duty diesel trucks. Lower cost sensors, if used in place of traditional research-grade analyzers, could enable wider application of this method, including use as a monitoring tool to identify high-emitting trucks that may warrant inspection and maintenance. However, low-cost sensors have for the most part only been evaluated under ambient conditions as opposed to source-influenced environments with rapidly changing pollutant concentrations. This study compared black carbon (BC) emission factors determined using different BC and carbon dioxide (CO_2_) sensors that range in cost from $200 to $20,000. Controlled laboratory experiments show that traditional zero and span steady-state calibration checks are not robust indicators of sensor performance when sampling short duration concentration peaks. Fleet BC emission factor distributions measured at two locations at the Port of Oakland in California with 16 BC/CO_2_ sensor pairs were similar, but unique sensor pairs identified different high-emitting trucks. At one location, the low-cost PP Systems SBA-5 agreed on the classification of 90% of the high emitters identified by the LI-COR LI-7000 when both were paired with the Magee Scientific AE33. Conversely, lower cost BC sensors when paired with the LI-7000 misclassified more than 50% of high emitters when compared to the AE33/LI-7000. Confidence in emission factor quantification and high-emitter identification improves with larger integrated peak areas of CO_2_ and especially BC. This work highlights that sensor evaluation should be conducted under application-specific conditions, whether that be for ambient air monitoring or source characterization.

## 1. Introduction

Heavy-duty diesel trucks transport consumer goods, commodities, agricultural products, and other materials that are essential to the economy. However, they are also significant emitters of nitrogen oxides (NO_x_), diesel particulate matter (DPM), and other pollutants that contribute to unhealthy levels of air pollution in many urban regions [1,2,3,4,5]. Environmental justice communities, including those near seaports, intermodal rail yards, and other industrial activity, are exposed to greater levels of truck pollution compared to those of higher socioeconomic status [6,7,8].

Emission standards for new trucks have resulted in the wide use of diesel particle filters (DPFs) to reduce tailpipe emissions of DPM, of which black carbon (BC) is major constituent, and selective catalytic reduction (SCR) systems to reduce tailpipe emissions of NO_x_ [9,10]. Studies of in-use heavy-duty diesel trucks in California have shown marked decreases in average BC emissions within a short span of time as fleets universally adopted DPFs [11,12,13,14]. However, a small percentage of in-use trucks in these fleets have BC emissions that are substantially higher than the average emission rate, and these high emissions may be due to deteriorating performance of aging particle filters [12,15,16]. For instance, the highest emitting 10% of a DPF-equipped drayage truck fleet at the Port of Oakland in California accounted for three-quarters of the fleet’s total BC emissions. This level of skewness in the distribution of emissions from in-use vehicles has been widely reported across various vehicle types and pollutants [12,13,17,18,19,20].

Presently, California is developing a heavy-duty truck inspection and maintenance program to ensure that emission control systems are properly functioning and trucks remain low-emitting throughout their entire operating life [21]. A screening tool, such as the exhaust plume capture method that measures emissions as on-road trucks pass by a fixed location, may be helpful to identify those highest-emitting trucks that most warrant inspection and possible maintenance. The method can efficiently quantify emissions from a large sample of trucks because of its non-invasive design, which does not require any installation of on-board measurement technologies. A key feature of this approach is that it would avoid all in-use trucks from having to report to a testing facility, as most trucks are relatively low-emitting and would not be flagged by this system.

Existing and emerging lower cost sensors could enable a wider use of the on-road exhaust plume capture method as a monitoring tool if used in place of research-grade analyzers that have been traditionally employed by both researchers and regulators [12,13,18,22]. In addition to their affordability, low-cost sensors are smaller and less power consuming than research-grade analyzers. For example, the California Air Resources Board is presently evaluating the use of low-cost BC and CO_2_ sensors to measure heavy-duty diesel truck BC emission factors [23]. However, low-cost sensors have primarily been evaluated as an economical tool to measure ambient levels of some pollutants; there has been little to no evaluation of the performance of low-cost sensors in an exhaust plume sampling application, where pollutant concentrations change rapidly from ambient concentrations [24,25,26,27,28,29]. If low-cost sensors are to be relied on to screen for high-emitting trucks, their performance should be thoroughly assessed for this specific application.

For these reasons, this study compared BC emission factors and the identification of high-emitting trucks when using different BC and carbon dioxide (CO_2_) sensors ranging in cost from a couple hundred to over twenty thousand dollars. Laboratory and field measurements reveal that different pairs of BC/CO_2_ sensors may greatly differ in their BC emission factor quantification and high emitter identification.

## 2. Materials and Methods

**Figure A1:**
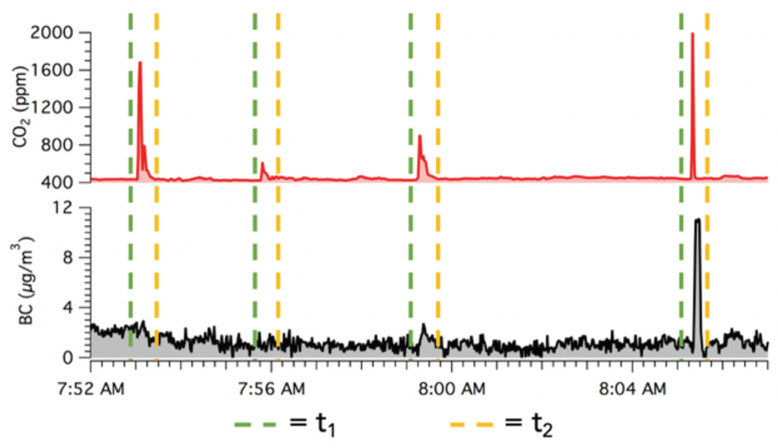
Concentration time series showing example peaks from roadside sampling of truck exhaust. The integration bounds (t_1_, t_2_) for determining fuel-based emission factors by the carbon balance method for each pollutant peak event are shown by dashed green and yellow lines. Minor tick marks show increments of 15 s.

This study evaluated the relative performance of four BC and five CO_2_ sensors under controlled laboratory conditions and during on-road sampling of truck exhaust. Table 1 lists these pollutant sensors, their approximate price, and manufacturer-specified performance metrics. All of these sensors operate with a measurement frequency of 1 Hz or faster, which is a critical feature to properly measure the pollutant concentration peaks that occur when truck exhaust plumes are briefly sampled (Figure A1). All four BC sensors are filter-based light absorption photometers: the research-grade Magee Scientific Aethalometer model AE33 and its no longer commercially available but widely used predecessor model AE16 (Berkeley, CA, USA), the AethLabs microAeth model MA300 (San Francisco, CA, USA), and the pre-commercial Aerosol Black Carbon Detector (ABCD), which UC Berkeley custom built and benchmarked against the AE33 [26]. All five CO_2_ sensors employ non-dispersive infrared spectroscopy and are listed in Table 1.

**Figure A2:**
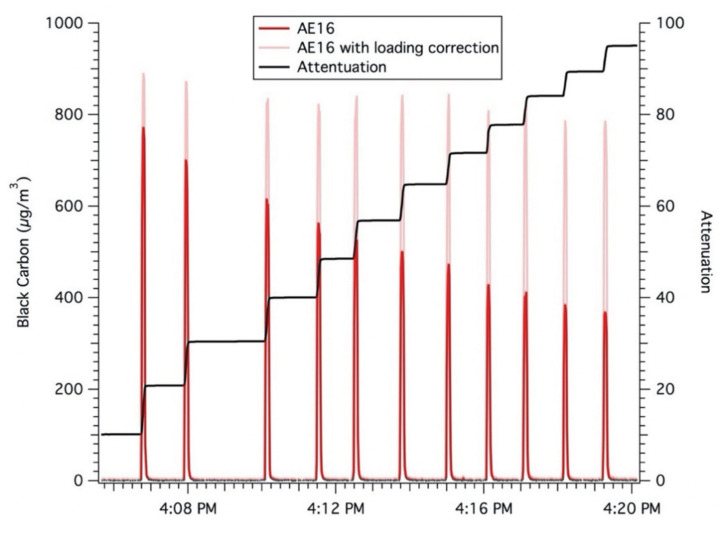
BC concentrations measured during controlled laboratory experiments with a steady emission source, with corresponding filter attenuation. After the AE16 data is post-processed using Equation (A1) (pink).

Prior to testing, CO_2_ sensors were calibrated with certified zero air and a 2000 ppm CO_2_ span gas. There is no analogous way to calibrate BC sensors; however, BC concentrations were adjusted for the filter-loading sampling artifact. The AE33 and MA300 have built-in software to perform a real-time adjustment, and BC concentrations from the AE16 and ABCD were manually post-processed (Equation (A1) and Figure A2) [30]. The analyzer-specific maximum filter loading was limited to optical attenuation values of ~70 to 100. Once that limit was reached, the AE33, AE16, and MA300 auto-advanced their filters, and the ABCD filter was manually changed.

**Figure A3:**
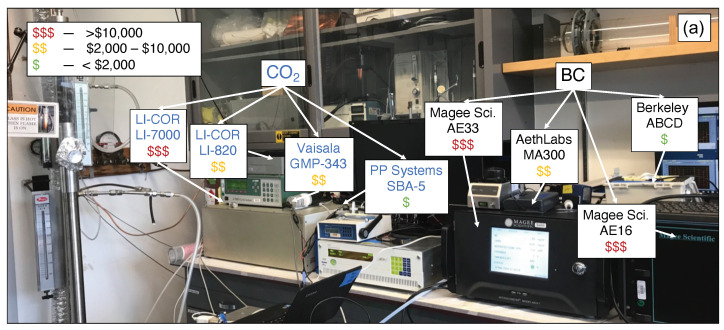

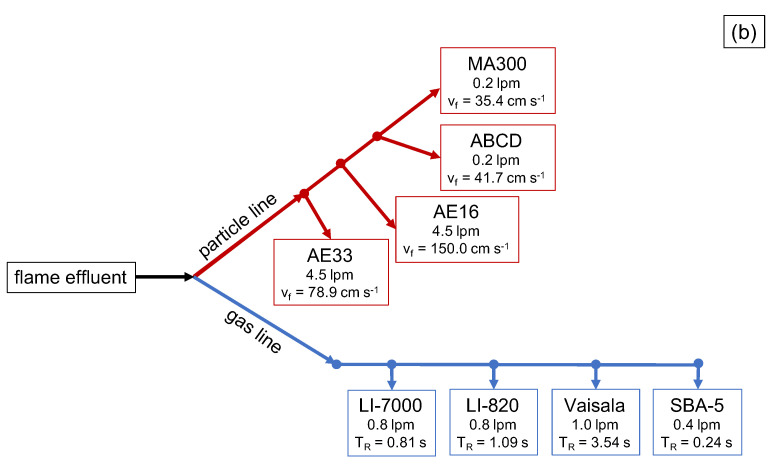
(**a**) Arrangements of sensors sampling the effluent of the inverted flame during laboratory testing. Price category scale is noted: $, low-cost (≤$2000); $$, mid-cost ($2000–$10,000); and $$$, high-cost (>$10,000). (**b**) Sampling configuration of sensors during laboratory testing. Flow rates of each sensor (lpm), residence times (T_R_) of the gas sample in the optical cell for CO_2_ sensors (s), and the filter face velocities (v_f_) for BC sensors (cm s^−1^) are shown in the figure.

An inverted methane/air flame that produces selectable and stable concentrations of BC and CO_2_ was used to simulate truck exhaust plume sampling in the laboratory [31]. By switching between room air and flame effluent, the sensors were exposed to short duration pollution events to mimic trucks passing. Five-second long peaks were generated periodically to allow time for concentrations to return to baseline values. Additionally, steady concentrations were sampled for longer durations of ~60 s to compare the dynamic responses of the instruments under conditions that are typical of calibration. Four CO_2_ and four BC sensors were arranged in the configuration shown in Figure A3a. Sensor sampling flow rates, optical cell residence times, and filter face velocities are reported in Figure A3b. The K30 CO_2_ sensor was not used during laboratory testing.

**Figure A4:**
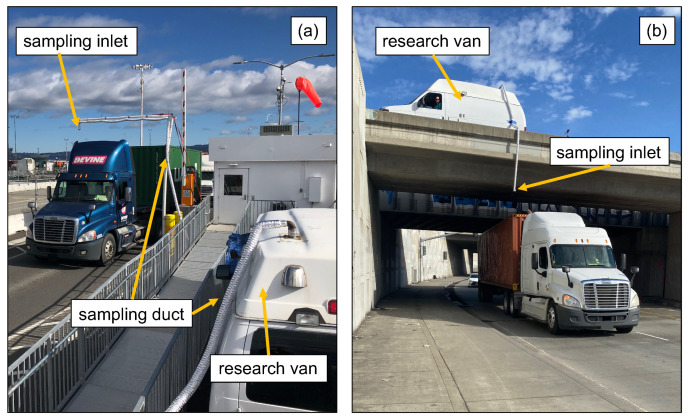
Field sampling of Port of Oakland drayage trucks at the (**a**) Terminal Entrance and on the (**b**) Arterial Road. The sampling inlet was aligned with truck’s right vertical exhaust stack and the sampling duct delivered sampled truck exhaust to the suite of sensors housed in the research van.

Tailpipe exhaust from DPF-equipped drayage trucks was measured at two locations at the Port of Oakland in California: at a major terminal entrance (Terminal Entrance) during five weekdays in March 2019 and on an arterial road (Arterial Road) that serves as a major access route to the Port of Oakland for seven weekdays in January–February 2020. At both locations, the exhaust from passing trucks was delivered to the suite of pollutant sensors housed in a research van via a flexible sampling duct that was closely aligned with the right vertical exhaust stack of passing trucks (Figure A4). At the Terminal Entrance, the sampling inlet was located ~3 m beyond the terminal’s check-in kiosk, where trucks came to a complete stop before entering the terminal. When driving under the sampling inlet, trucks were gently accelerating from the complete stop and moving ~8 km h^−1^ (~2.2 m s^−1^). At this location, trucks passed by one at a time about once per minute in a single roadway lane. At the Arterial Road, a site previously used for on-road sampling, the research van was located on an overpass, and the sampling duct was oriented above trucks passing in the right-most roadway lane [11,12]. Trucks passed the sampling inlet either after accelerating from a stop at a traffic signal ~50 m before the sampling point or at a cruising speed of ~50 km h^−1^ (~14 m s^−1^).

Fuel-based black carbon emission factors (EF_BC_) are calculated via a carbon mass balance method using Equation (1) [32]. The background-subtracted concentration time series of BC and CO_2_ are integrated between the start and end time (t_1_, t_2_) of an exhaust plume, which typically lasts around 5–10 s (Figure A1). Negative, near-zero emission factors can occur in cases of small or no BC response during a plume event. The ratio of the integrated areas is multiplied by the molecular weight ratio of CO_2_ to carbon (44/12) and the weight fraction of carbon in diesel (w_c_ = 0.87), assuming the complete conversion of carbon in the fuel to carbon dioxide during combustion [32]. The factor of 10^3^ converts EF_BC_ to units of grams of BC emitted per kilogram of fuel burned (g kg^−1^).

(1) $${\mathrm{EF}}_{\mathrm{BC}}\;=\;\frac{\int_{t_{1}}^{t_{2}}[\mathrm{BC}(t)-\mathrm{BC}(t_{1})]\mathrm{dt}}{\int_{t_{1}}^{t_{2}}[{\mathrm{CO}}_{2}(t)-{\mathrm{CO}}_{2}(t_{1})]\mathrm{dt}}\;\times \;\frac{44}{12}\;\times \;w_{c}\;\times \;10^{3}$$

## 3. Results and Discussion

### 3.1. Sensor Performance in the Laboratory

Laboratory testing consisted of four separate experiments with different pollutant concentrations and corresponding BC emission factors, as reported in Table 2. For each experiment, sensors were tested with 50 identical simulated exhaust plume samples and one 60 s steady-state emissions period. For reference, peak CO_2_ and BC concentrations encountered when measuring on-road truck exhaust plumes typically range from ~450 to 2000 ppm CO_2_ and from near-zero up to several hundred μg m^−3^ BC. All laboratory experiments fall within peak CO_2_ concentrations that have been measured during truck exhaust plume sampling. The same is true for BC concentrations during Experiments 0 and 1, but peak BC concentrations during Experiments 2 and 3 are in excess of what would be expected during truck exhaust plume sampling based on the work of the authors.

**Figure 1:**
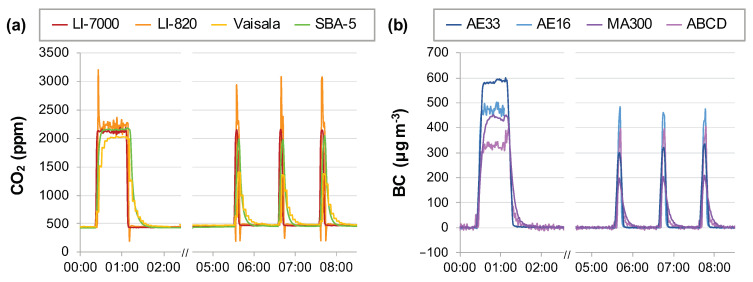
Example concentration time series from laboratory experiments. The time series show the varied responses of the (**a**) CO_2_ and (**b**) BC sensors tested in this experiment during steady-state and peak concentration events. For example, the higher cost LI-7000 accurately responds to the initial step change in CO_2_ concentration, while the lower cost LI-820 initially overshoots to ~3200 ppm before returning to the concentration plateau at ~2100 ppm.

An example of the response of the instruments during steady-state and peak concentration events can be seen in Figure 1. While all four CO_2_ sensors ultimately achieve the same steady-state value during the 60 s plateau (2100 ± 100 ppm CO_2_), the dynamic responses of the sensors differ (Figure 1a). The LI-7000 and LI-820 are the quickest to reach the plateau and to return to baseline. The LI-7000 concentration reflects the step change in the actual CO_2_ concentration, but the LI-820 briefly overshoots the actual plateau concentration and later dips below baseline at the end of the plateau. The SBA-5 and Vaisala were slower and slowest, respectively, to reach the steady-state plateau value, and their return to baseline was sluggish by comparison to the LI-COR analyzers. Unlike the Vaisala, whose delayed response can be attributed to the ~4 times larger residence time of air in its optical cell, the slow response of the SBA-5 is unexpected, given that the residence time of air in its optical cell was the shortest among the sensors tested (Figure A3b). In addition, the point-to-point variability was smallest for the SBA-5 data, indicating that a running average is internally applied to the reported data.

The different dynamic response of each sensor causes the relative appearances of the short duration peaks (5–20 s depending on instrument) to be very different (Figure 1a). More importantly, the integrated areas of the peaks are different. Across all laboratory experiments, the LI-820 overshoot of actual concentration causes the peak area to be overstated by 13–19% compared to the LI-7000. A consequence of overstating the peak area is understating the BC emission factor (Equation (1)), which has previously been reported during on-road truck exhaust plume measurements [11]. The areas of the shorter and broader peaks recorded by the SBA-5 and Vaisala are on average 52% and 5% greater than the peak areas recorded by the LI-7000 during the experiments. All of these differences illustrate that sensor response to steady-state or slowly changing concentrations (e.g., calibration or ambient air concentrations) may not be a good predictor of performance during short duration peak events.

Unlike the four CO_2_ sensors, the four BC sensors did not measure the same plateau value during the steady-state event shown in Figure 1. The AE16 and MA300 measured ~450 μg m^−3^, whereas the AE33 and ABCD recorded concentrations that were 150 μg m^−3^ higher and lower, respectively. Similarly, the areas of the BC peaks recorded by the AE16 and MA300 were similar, despite the smoother and broader MA300 peaks that indicate the analyzer applies a running average. Whereas the AE33 plateau value was ~33% larger than the steady-state values measured by the AE16 and MA300, the AE33 peak areas were only ~10% larger for this experiment. Meanwhile, the ABCD steady-state value was ~33% lower than the steady-state values measured by the AE16 and MA300, the ABCD peak areas were ~10% larger and about the same as the AE33 peak areas.

Interpreting the relative responses of BC sensors is complicated by the filter loading artifact, which is not a factor for the CO_2_ measurements. The observed differences in performances may be a consequence of the applied loading artifact corrections, which may be imperfect under the experimental conditions. 

Table 2 reports emission factor means (μ) and coefficients of variations (CV = |σ/μ|, where σ is the standard deviation) determined from specified pairs of BC/CO_2_ sensors for the four laboratory experiments. The CV represents the precision with which each sensor pair measured the BC emission factors across the 50 identical simulated exhaust plume samples.

**Figure A5:**
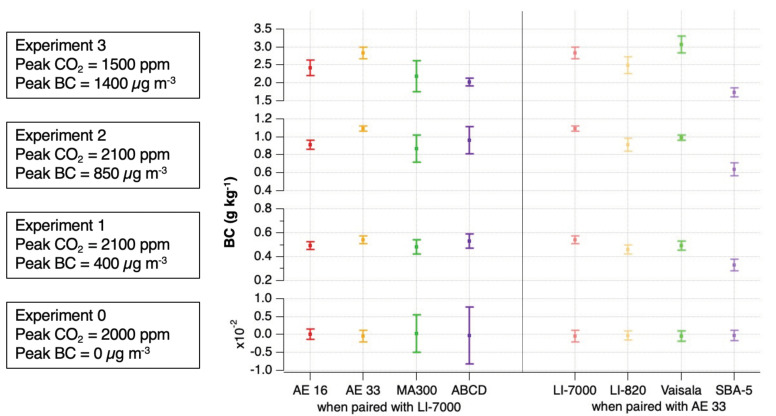
Average value (±1 standard deviation) of emission factors determined from laboratory experiments that targeted BC emission factors of ~0, ~0.5, ~1, and ~2 g kg^−1^ (Experiments 0–3, respectively and from bottom to top plots). The left panel shows the results of emission factors calculated with the specified BC sensors when paired with the LI-7000, and the right panel shows data from the specified CO_2_ sensors when paired with the AE33.

Variation in the precision of BC emission factors measured with the LI-7000 CO_2_ analyzer and four different BC sensors (top half of Table 2, Figure A5) can be attributed to differences in the performance of the BC sensors, as they all share the same CO_2_ analyzer. Non-zero emission factors were most precisely measured with the AE33 (3–6% CV), followed by the AE16 (5–7% CV), ABCD (5–17% CV), and the MA300 (13–20% CV). The AE33 also measured near-zero BC emission factors more precisely than the AE16, MA300, and ABCD, whose relatively noisier point-to-point baseline response yielded CV values ~8 times higher. Of the BC emission factors measured with the AE33 and four CO_2_ sensors (bottom half of Table 2, Figure A5), the research-grade LI-7000 yields the most precisely measured emission factors (3–6% CV). Switching among the four CO_2_ sensors does not vary the precision of measuring emission factors as much as observed between various BC sensors when paired with the LI-7000.

### 3.2. Sensor Performance in the Field

Truck BC emission factors measured at the Terminal Entrance (average EF ± 95% confidence interval = 0.10 ± 0.01 g kg^−1^, *n* = 843) were lower than at the Arterial Road (0.24 ± 0.04 g kg^−1^, *n* = 804), as summarized in Table A1. This difference is attributed to significantly lower speeds and engine loads at the Terminal Entrance compared to the Arterial Road.

In the present study, we treat the highest cost, research-grade AE33/LI-7000 sensor combination as the gold standard to which other sensor pairs are compared, which is reasonable given the results of the laboratory evaluations. The absolute value percent error (AVPE) in emission factors measured using alternate BC/CO_2_ sensor pairs compared to the AE33/LI-7000 standard is calculated as:

(2) AbsoluteValuePercentError(AVPE)of PairX=100× ∣(EFfromPairX)−(EFfromAE33/LI−7000)(EFfromAE33/LI−7000)∣.

**Figure 2:**
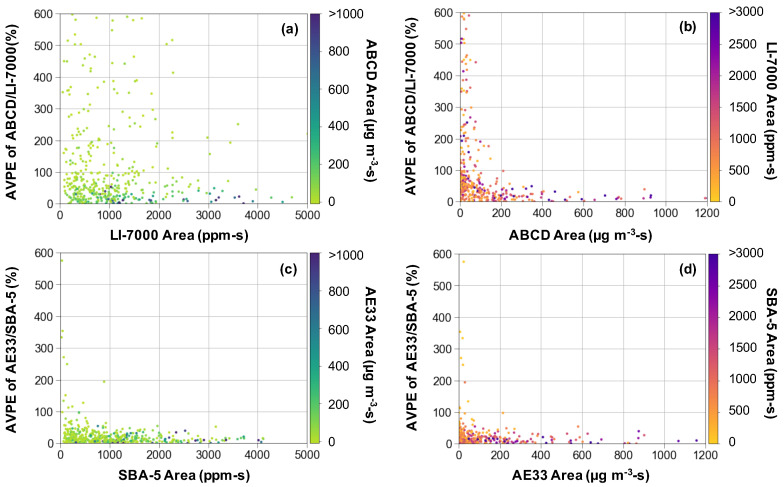
Absolute value percent error (AVPE) of emission factors measured using the ABCD/LI-7000 (**a**,**b**) and AE33/SBA-5 (**c**,**d**) as compared to emission factors measured using the AE33/LI-7000. Error is plotted versus integrated areas for the: (**a**) LI-7000, (**b**) ABCD, (**c**) SBA-5, and (**d**) AE33. Data shown are from measurements made at the Arterial Road in 2020.

In Figure 2, AVPEs are plotted versus the integrated peak areas of CO_2_ and BC from individual trucks for two sensor pairs as illustrative cases. The peak CO_2_ area is a measure of the strength of the exhaust plume capture. A larger area means that less dilution of the exhaust plume occurred before it was sampled. The peak BC area further depends on the formation of soot during combustion and the degree to which the soot is removed by an installed diesel particle filter. The error in ABCD/LI-7000 emission factors is shown as a function of LI-7000 peak CO_2_ area (Figure 2a) and ABCD peak BC area (Figure 2b), and the error in AE33/SBA-5 emission factors is shown as a function of SBA-5 CO_2_ and AE33 BC peak areas (Figure 2c,d, respectively).

**Figure A6:**
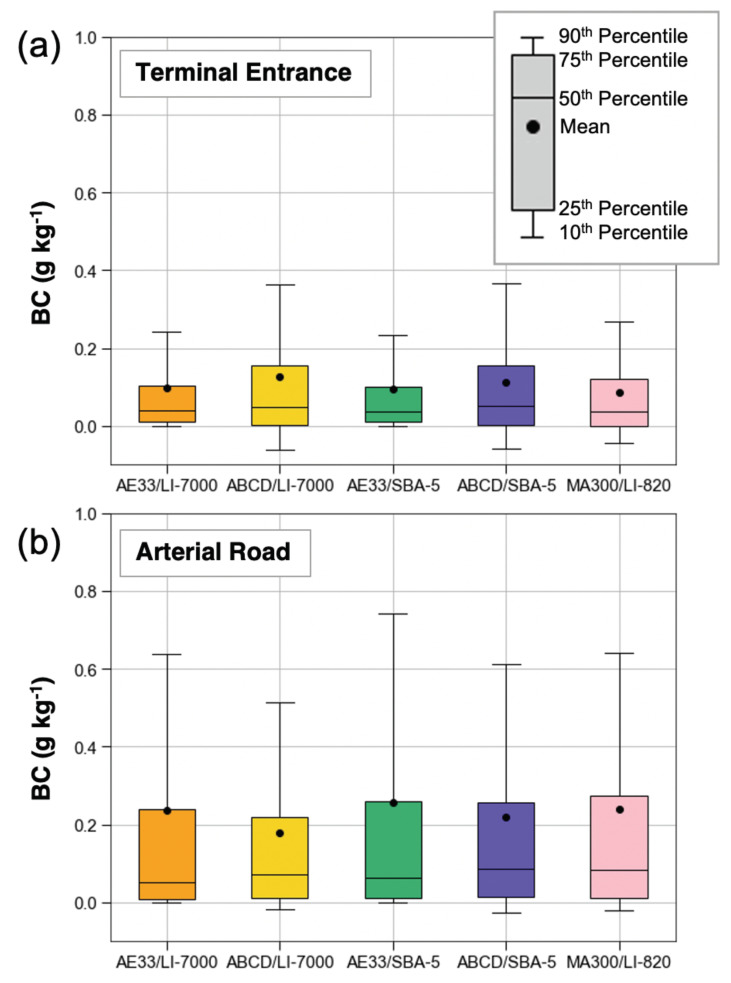
BC emission factors distributions measured at the (**a**) Terminal Entrance and (**b**) Arterial Road for five different sensor pairs.

Although the distributions of emission factors measured with different pairs of sensors are generally similar overall (Figure A6), the percent difference in emission factors for individual trucks can be very large. BC emission factors measured using the ABCD and AE33 can be up to 600% different. Some errors are even larger but are not shown in order to limit the bounds of the vertical axis. The error is smaller for larger BC peak areas, and it is independent of the strength of the CO_2_ plume capture; the darker data points that represent larger BC areas span the range of CO_2_ peak areas in Figure 2a. In contrast, the difference between emission factors measured with two different CO_2_ sensors is not nearly as large as that when measuring with two different BC sensors (Figure 2b vs. Figure 2a). The errors in emission factors measured with the AE33 paired with the SBA-5 rather than the LI-7000 (Figure 2c,d, respectively) are mostly within 50% rather than within 600%.

**Figure A7:**
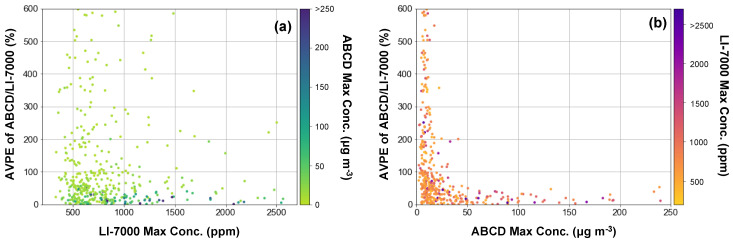
Percent error of emission factors measured using the ABCD/LI-7000 as compared to emission factors measured using the AE33/LI-7000, plotted versus (**a**) LI-7000 CO_2_ and (**b**) ABCD BC peak maximum concentrations. Data shown are from measurements made at the Arterial Road in 2020.

The emission factor measurement discrepancy is much more dependent on the BC peak area rather than the strength of the exhaust plume capture (i.e., the CO_2_ integrated area). There is a steep reduction in error as the integrated peak area increases for BC (Figure 2b) compared to the less defined improvement for increasing CO_2_ peak area (Figure 2a). Due to the steepness of the trend in Figure 2b, it is possible to identify a threshold below which errors become unacceptably large related to the gold standard. For example, when ABCD peak areas are below ~100 μg m^−3^-s or when the peak BC concentration is less than ~30 μg m^−3^ (Figure A7), the difference in BC emission factors measured using the ABCD and AE33 often exceeds 50%.

### 3.3. Field Performance of Sensors Identifying High Emitters

**Figure 3:**
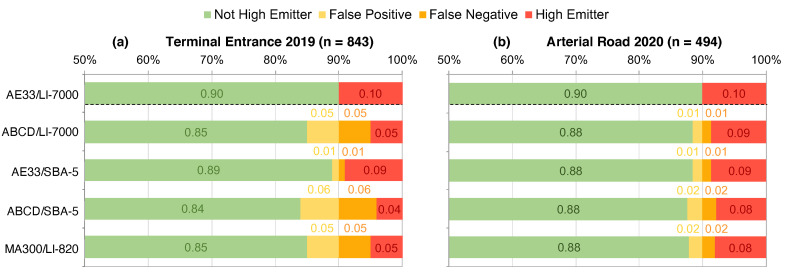
Agreement of high emitter classification across three different sensor pairs for the (**a**) Terminal Entrance and (**b**) Arterial Road fleets. Classifications are compared to the AE3/LI-7000, and labeled as a high emitter (red) or not a high emitter (green) as well as a false positive (yellow) or negative (orange). Each bar adds up to 100% of the sampled fleet.

To evaluate the agreement of high-emitting trucks identified using different pairs of BC/CO_2_ sensors, the research-grade AE33/LI-7000 pair is again assumed to be the gold standard to which classifications by other sensor pairs are compared. Note that different criteria can be chosen to establish a threshold emission factor for the purpose of classifying a truck as a high emitter. In this analysis, we defined the high emitter criteria to be the highest 10% of all measured BC emission factors in a fleet and used the 90th percentile emission rate as the high emitter threshold. Figure 3 categorizes individual trucks as high emitters, false positives, false negatives, or not high emitters. A false positive means that the specified sensor pair identified a truck as a high emitter but that truck was not a high emitter according to the AE33/LI-7000 pair, and vice versa for a false negative.

**Figure A8:**
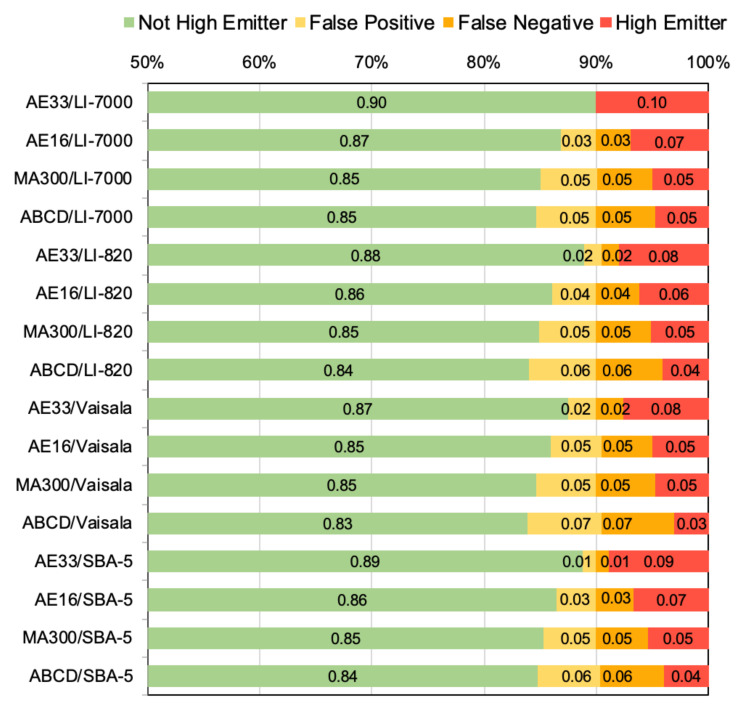
Agreement of high emitter classification across all sensor pairs for the Terminal Entrance fleet. Relative to the highest-emitting 10% of each fleet identified by the highest-cost pair, the AE33/LI-7000, emission factor distributions for alternative sensor pairs are categorized by those trucks that are also identified as high-emitting (red) and not (green), versus those that disagree and are false positives (yellow) or false negatives (orange). Each bar adds up to 100% of the sampled fleet. Due to rounding, the sum of high emitters and false positives and false negatives may not equal 10%.

At the Terminal Entrance, the combination of the high-cost AE33 and low-cost SBA-5 showed the best agreement to the gold standard; one out of every 10 trucks identified as a high emitter by this pair was identified as a clean truck by the AE33/LI-7000 (i.e., 10% misclassification). This contrasts the poor performance of the SBA-5 during the laboratory experiments noted above. Improvement in SBA-5 performance during field sampling may be due to the mostly lower CO_2_ peak concentrations observed in the field compared to laboratory experiments (Table 2 and Table A1). The other sensor pairs, the ABCD/LI-7000, ABCD/SBA-5, and MA300/LI-820, misclassified 50–60% of their high emitters. Across all 16 sensor pairs, emission factors calculated with the AE33 agree within 20% of the gold standard regardless of CO_2_ sensor, whereas the mid- and low-cost BC sensors misclassify more than half of their high emitters when paired with the high-cost LI-7000 (Figure A8). This larger degree of misclassification introduces considerable uncertainty in the identification of high emitters, where the uncertainty is driven by the differences in BC sensor performance.

**Figure A9:**
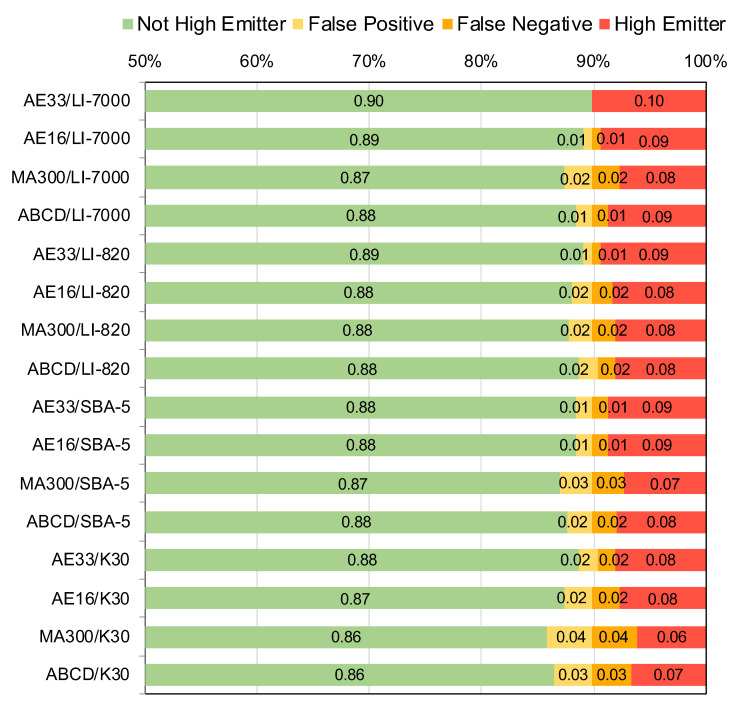
Agreement of high emitter classification all sensor pairs for the Arterial Road fleet. Relative to the highest-emitting 10% of each fleet identified by the highest-cost pair, the AE33/LI-7000, emission factor distributions for alternative sensor pairs are categorized by those trucks that are also identified as high-emitting (red) and not (green), versus those that disagree and are false positives (yellow) or false negatives (orange). Each bar adds up to 100% of the sampled fleet. Due to rounding, the sum of high emitters and false positives and false negatives may not equal 10%.

At the Arterial Road, where the exhaust plume capture was stronger and peak concentrations were larger, there is more consistency in high-emitter classification across all sensor pairs (Table A1). Most pair classifications were within 20% agreement with the gold standard, and at worst, the MA300/K30 misclassified 40% of their high emitters (Figure A9).

**Figure 4:**
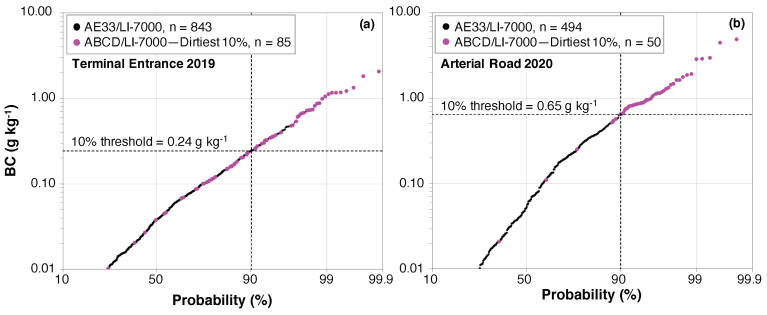
Probability distributions of emission factors determined with the AE33/LI-7000 for the (**a**) Terminal Entrance and (**b**) Arterial Road fleets (black dots). The dashed lines indicate the AE33/LI-7000 threshold for classifying the dirtiest 10% of the fleet as high emitters, those located in the upper right quadrant. The high-emitting trucks identified by the ABCD/LI-7000 are highlighted on these distributions (fuchsia dots). Note that these superimposed points are AE33/LI-7000 emission factor values for high-emitting trucks (dirtiest 10%) according to the ABCD/LI-7000.

In addition to evaluating the frequency of high emitter misclassification, we further consider the discrepancy in the magnitude of the emission factors when there are misclassifications. Figure 4 shows the probability distributions of BC emission factors determined with the AE33/LI-7000 at the Terminal Entrance and Arterial Road (black dots), with the dirtiest 10% of each fleet falling to the right of the vertical dashed lines and classified as high emitters. The fuchsia dots are AE33/LI-7000 emission factor values for high-emitting trucks (dirtiest 10%) identified by the ABCD/LI-7000. Those fuchsia dots in the upper right quadrant of each plot—above the 10% threshold line and to the right of the 90% probability line—are high emitters identified by both the AE33 and ABCD when paired with the LI-7000; those fuchsia dots in the lower left quadrant are false positives, where the ABCD/LI-7000 identified the truck as a high emitter but the AE33/LI-7000 did not.

At the Terminal Entrance, many emission factors that the ABCD/LI-7000 identified as high-emitting were very clean according to the research-grade AE33/LI-7000. The high-emitting 10% of trucks measured using the ABCD/LI-7000 had BC emission factors >0.39 g kg^−1^. According to the AE33/LI-7000, these same trucks had emission factors spanning two orders of magnitude, ranging from 0.01 g kg^−1^ to >1 g kg^−1^. At the Arterial Road, not only was there a lower rate of high emitter misclassification, but the majority of ABCD/LI-7000 false positives were also just slightly below the threshold established by the AE33/LI-7000, with only a few egregious outliers. Similar to the results above, this difference by location is likely due to the stronger plume capture and higher peak concentrations, especially BC concentrations, measured at the Arterial Road, which are conditions that improve analyzers’ performance and agreement across sensor pairs.

**Figure 5:**
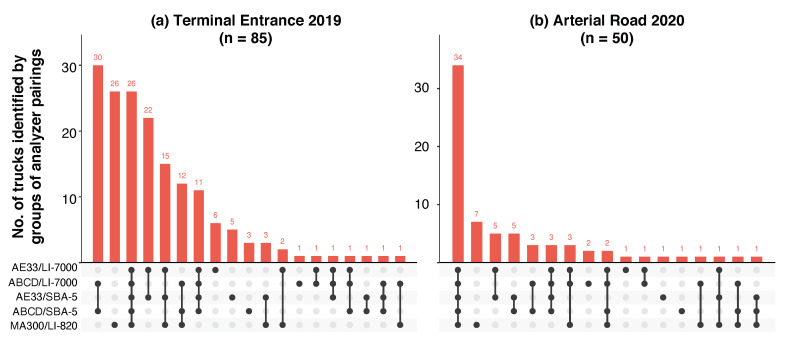
Intersections of high-emitter occurrences across five different sensor pairs measured at the (**a**) Terminal Entrance and (**b**) Arterial Road. Here, sensor pairs that identify the same high emitters are vertically marked with dots, and the number of occurrences in that intersection is shown as a vertical bar. Each unique intersection is plotted on the horizontal axis, from highest number of occurrences to least.

Figure 5 shows the intersections of high emitter occurrences across five different sensor pairs. Intersections where sensor pairs identify the same high emitters are vertically marked with lines that connect dots that correspond to sensor pairs, and the number of occurrences in that intersection is shown as a vertical bar. Each unique intersection is plotted on the horizontal axis from the highest to lowest number of occurrences. Of the 85 high emitters identified by each unique pair as the highest emitting 10% of the Terminal Entrance fleet, only 26 (31%) were shared across all five sensor pairs. Although the number of commonly identified high emitters is low, the intersection adds confidence in flagging those 26 trucks as truly high-emitting. Given that it was found that the imprecision of BC sensors is greater than the CO_2_ sensors tested in this study, it stands to reason that intersections that involve different BC sensors add a greater degree of confidence than intersections that involve the same BC sensor and different CO_2_ sensors. For instance, 30 high-emitting trucks were identified by both the ABCD/SBA-5 and ABCD/LI-7000 pairs at the Terminal Entrance. However, this overlap does not add as much confidence in the classification as compared with that from the intersection of three different BC analyzers (AE33, MA300, and ABCD) for 26 trucks. Following the results from above, there was more agreement at the Arterial Road, with 85% of high emitters (34 out of 50 trucks) shared across all five sensor pairs. This highlights the significance of sampling site selection.

## 4. Conclusions

While California is presently defining an inspection and maintenance program to reduce emissions from high-emitting vehicles, small numbers of high-emitting vehicles contribute the majority of emissions around the world. The use of lower cost sensors as part of an on-road screening tool could increase the efficiency of identifying these highest-emitting vehicles. This study finds that different grades of CO_2_ and especially BC sensors do not equally respond to concentration peaks encountered during exhaust plume sampling, leading to inaccurately measured emission factors, and consequently false positive and false negative high emitter identification. Here, the high-emitting threshold was defined as the 90th percentile of fleet emission factor distribution; other definitions may lead to somewhat different levels of agreement between sensor pairs than those reported above. Confidence can be increased by selecting sampling locations where driving conditions result in high BC emissions or by using more than one BC sensor, if not too impractical. This study also identified that a low-cost CO_2_ sensor (e.g., the low-cost SBA-5) may serve as an adequate substitute for research-grade analyzers that cost ~10 times more, with a low rate of misclassification of high-emitting trucks. Though this is not an exhaustive evaluation of any of the sensors used, it does highlight that sensors should be evaluated under application-specific conditions, whether that be for ambient air monitoring or source characterization. This is a cautionary message for the growing interest in using existing and emerging low-cost sensors.

## Figures and Tables

**Table 1:** Black carbon (BC) and carbon dioxide (CO_2_) sensors used in this study, their approximate price ($K USD), and manufacturer-specified performance metrics.

	Make and Model	Approximate Price ($K)	Accuracy *^,^^	Precision, Sensitivity, or Noise ^^^
**CO_2_**	CO2Meter K30	0.2	±30 ppm ± 3%	±20 ppm ± 1%, Sensitivity
	PP Systems SBA-5	2	<1%	N/A
	Vaisala GMP343	3	± (5 ppm + 2%)	±3 ppm at 370 ppm, Noise
	LI-COR LI-820	4	3%	<1 ppm at 370 ppm, RMS Noise
	LI-COR LI-7000	12	1%	12 ppb at 370 ppm, RMS Noise
**BC**	UC Berkeley ABCD	N/A	~25% relative to AE33	~9%, Precision
	AethLabs MA300	10	N/A	N/A
	Magee Scientific AE16	20	5%	< 0.1 μg m^−3^, Sensitivity
	Magee Scientific AE33	25	N/A	N/A

* Performance metrics specified by the manufacturer except for the pre-commercial Aerosol Black Carbon Detector (ABCD), which was developed and characterized by researchers at the University of California, Berkeley [26]. ^ N/A notes when price, accuracy, precision, sensitivity, or noise was not listed by manufacturer. RMS is root mean squared.

**Table 2:** Emission factor means (μ, g kg^−1^) and coefficients of variation (CV, %) based on the combination of measurements from the specified BC and CO_2_ analyzers.

**Specified BC Sensor Paired with LI-7000 CO_2_ Analyzer, μ (CV)**					
**Exp.**	**Peak BC** **(μg m^−3^)**	**AE33**	**AE16**	**MA300**	**ABCD**
0	0	−0.41 × 10^−3^ (381)	0.07 × 10^−3^ (2213)	0.23 × 10^−3^ (2216)	−0.31 × 10^−3^ (2519)
1	450	0.54 (6.3)	0.49 (5.3)	0.48 (13.4)	0.53 (10.8)
2	850	1.08 (3.3)	0.91 (6.0)	0.86 (17.2)	0.96 (16.6)
3	1400	2.85 (4.9)	2.39 (6.6)	2.27 (19.9)	2.04 (4.8)
**Specified CO_2_ Sensor Paired with AE33 BC Analyzer, μ (CV)**					
**Exp.**	**Peak CO_2_** **(ppm)**	**LI-7000**	**LI-820**	**Vaisala**	**SBA-5**
0	2000	−0.41 × 10^−3^ (381)	−0.36 × 10^−3^ (345)	−0.38 × 10^−3^ (374)	−0.36 × 10^−3^ (392)
1	2100	0.54 (6.3)	0.46 (8.7)	0.49 (7.2)	0.34 (7.1)
2	2100	1.08 (3.3)	0.91 (8.1)	0.99 (3.6)	0.64 (12.1)
3	1500	2.85 (4.9)	2.53 (7.5)	3.11 (6.9)	1.73 (5.9)

## Appendix A

The plume capture method calculates fuel-based emission factors via the carbon balance method. Background-subtracted concentration time series of black carbon (BC) and carbon dioxide (CO_2_) are integrated between the start and end time (t_1_, t_2_) of an exhaust plume (Figure A1). Figure A1 shows an example concentration time series for BC and CO_2_ from the roadside sampling of trucks. The vertical dashed lines represent the integration bounds used to calculate an emission factor. CO_2_ and BC peak magnitudes vary based on the amount of dilution that occurs before the plume capture and the engine production and diesel particle filter removal of BC. All of these sensors in this study operate with a measurement frequency of 1 Hz or faster, which is a critical feature to properly measure the pollutant concentration peaks that lasts around 5–10 s.

The loading correction for the AE16 and the ABCD was applied using Equation (A1), with the correction factor, a = 0.64. Figure A2 shows simulated plume events during laboratory experiments, as measured by the AE16, which does not have a real-time correction. The corresponding filter attenuation of AE16 is also shown. The AE16 BC concentration that has been post-processed for the loading artifact correction is plotted in pink.

(A1) $${\mathrm{BC}}_{\mathrm{corr}}\;=\;\frac{\mathrm{BC}}{a\cdot e^{-\frac{\mathrm{ATN}}{100}}+(1-a)}$$

During laboratory experiments, four CO_2_ and four BC sensors were arranged in the configuration shown in Figure A3. Five-second long peaks, mimicking trucks passing, were generated periodically to allow time for concentrations to return to baseline values. In addition, steady-state concentration events lasting ~60 s were performed to compare the dynamic responses of the instruments.

Drayage truck tailpipe exhaust emissions were measured at two locations at the Port of Oakland in California: at a major terminal entrance (Terminal Entrance) during five weekdays in March 2019 and on an arterial road (Arterial Road) that serves as a major access route to the Port of Oakland for seven weekdays in January–February 2020. At both locations, the exhaust from passing trucks was delivered to the suite of pollutant sensors housed in a research van via a flexible sampling duct that was closely aligned with the right vertical exhaust stack of passing trucks (Figure A4).

## Appendix B

Variation in the precision of BC emission factors measured with the four different CO_2_ sensor and four different BC sensors can mostly be attributed to differences in the performance of the BC sensors. In Figure A5, the variabilities, shown by the standard deviation bars, are more variable across BC sensors paired with the LI-7000 than CO_2_ sensors paired with the AE33.

Truck BC emission factors measured at the Terminal Entrance (average EF ± 95% confidence interval = 0.10 ± 0.01 g kg^−1^, *n* = 843) were lower than at the Arterial Road (0.24 ± 0.04 g kg^−1^, *n* = 804), as summarized in Table A1.

**Table A1:** Summary of BC emission factors and peak CO_2_ and BC concentrations at the Terminal Entrance 2019 and Arterial Road 2020 sampling locations measured with the AE33 and LI-7000.

	Sample Statistic	Terminal Entrance 2019	Arterial Road 2020
**BC Emission Factors** (**g kg^−1^**)	Mean (± 95% Confidence)	0.10 ± 0.01	0.24 ± 0.04
	Median	0.04	0.05
	90th Percentile	0.24	0.65
	10th Percentile	0.00	0.00
**Absolute; Background-Subtracted** **Peak CO_2_** (**ppm**)	Mean	695; 240	850; 400
	Median	620; 180	720; 260
	90th Percentile	1070; 550	1375; 840
	10th Percentile	470; 55	515; 150
**Absolute;** **Background-Subtracted** **Peak BC** (**µg m^−3^**)	Mean	3.8; 3.4	23.2; 18.0
	Median	1.7; 1.5	8.8; 4.9
	90th Percentile	6.7, 5.5	45.7; 35.7
	10th Percentile	0.9; 1.3	3.2; 1.8

At both locations, the distributions of emission factors measured with different pairs of sensors are generally similar overall, although differences were found in the absolute value percent errors between sensor pairs and the AE33/LI-7000 sensor pairs. This also resulted in a misclassification of high emitters.

Compared to a very steep reduction in error as the integrated peak area increases for BC (Figure 2b), the improvement is less defined for the increasing CO_2_ peak area (Figure 2a). Due to the steepness of the trend in Figure 2b, it is possible to identify a threshold below which errors become unacceptably large. For example, as shown in Figure 2 and Figure A7, when BC peak areas are below ~100 μg m^−3^-s or when the peak BC concentration is less than ~30 μg m^−3^, the difference in BC emission factors measured using the ABCD and AE33 often exceeds 50%. Therefore, the emission factor measurement discrepancy is much more dependent on the BC peak area than the strength of the exhaust plume capture (i.e., the CO_2_ integrated area).

Across all 16 sensor pairs, emission factors measured at the Terminal Entrance and calculated with the AE33 agree within 20% of the gold standard regardless of CO_2_ sensor, whereas the mid- and low-cost BC sensors misclassify more than half of their high emitters when paired with the high-cost LI-7000 (Figure A8). At the Arterial Road, exhaust plume capture was stronger and peak concentrations were larger; there is more consistency in high-emitter classification across all sensor pairs, with most agreeing within 20% of the gold standard. At worst, the MA300/K30 misclassified 40% of their high emitters (Figure A9).
